# Data Sharing While Using a Closed-Loop System: Qualitative Study of Adolescents' and Parents' Experiences and Views

**DOI:** 10.1089/dia.2020.0637

**Published:** 2021-06-29

**Authors:** Julia Lawton, Ruth I. Hart, Barbara Kimbell, Janet M. Allen, Rachel Elizabeth Jane Besser, Charlotte Boughton, Daniela Elleri, Julia Fuchs, Atrayee Ghatak, Tabitha Randell, Ajay Thankamony, Nicola Trevelyan, Roman Hovorka, David Rankin

**Affiliations:** ^1^ Usher Institute, Medical School, University of Edinburgh, Edinburgh, United Kingdom.; ^2^ Wellcome Trust–Medical Research Institute of Metabolic Science, University of Cambridge, Cambridge, United Kingdom.; ^3^ Department of Paediatrics, University of Cambridge, Cambridge, United Kingdom.; ^4^ NIHR Oxford Biomedical Research Centre, Oxford University Hospitals NHS Foundation Trust, Oxford, United Kingdom.; ^5^ Department of Paediatrics, University of Oxford, Oxford, United Kingdom.; ^6^ Royal Hospital for Sick Children, Edinburgh, United Kingdom.; ^7^ Alder Hey Children's NHS Foundation Trust, Liverpool, United Kingdom.; ^8^ Nottingham Children's Hospital, Nottingham, United Kingdom.; ^9^ Children's Services, Cambridge University Hospitals NHS Foundation Trust, Addenbrookes Hospital, Cambridge, United Kingdom.; ^10^ Department of Child Health, Southampton Children's Hospital, Southampton, United Kingdom.

**Keywords:** Closed-loop system, Remote monitoring, Data sharing, Adolescents, Parents, Diabetes

## Abstract

***Objective:*** To understand and explore data sharing practices among adolescents and their parents using a closed-loop system.

***Methods:*** Eighteen adolescents (aged 11–18 years) and 19 parents were interviewed after adolescents had ∼6 months experience of using a closed-loop system, which permitted them to share glucose and insulin data with parents/caregivers. Data were analyzed thematically.

***Results:*** There was considerable variability in how parent–child dyads perceived, valued, and undertook data sharing. Parents of early adolescents (11–13 years) reported making extensive use of “real time” data to remotely manage their child's diabetes and early adolescents described needing and wanting this input. Parents of middle adolescents (14–16 years) described making greater use of retrospective data. To avoid conflict and encourage and support their son/daughter's autonomy, these individuals reported practicing watchful waiting and only intervening after concerns about a pattern of problematic behavior or their child's safety arose. Middle adolescents indicated that data sharing had been done primarily for the benefit of their parents, although they also noted quality of life benefits for themselves. Among late adolescents (17+ years), parents were simply remote because their son/daughter had not permitted access to their data. Participants recommended clear ground rules be put in place about when, and how, data sharing should be used.

***Conclusions:*** To help parent–child dyads use data sharing in ways which minimize conflict and optimize constructive parental support, we recommend tailored input and support, which takes account of family dynamics, the young person's developmental maturity, and the different ways in which data are used across the adolescent age range.

## Background

A continuous glucose monitor (CGM) measures interstitial glucose continuously and transmits the data to the user's display device (e.g., a smart device or pump). CGM technology also includes software which enables users to analyze their data and look for patterns and trends to inform insulin dose adjustments. Recent developments now permit users to share their data with informal caregivers, such as parents and partners, who can check/review glucose data on their own smart devices (e.g., smartphone) and receive alerts when hypoglycemia and hyperglycemia occur.

To date, limited research has looked at young people's and parents' experiences of CGM data sharing. This research has mostly reported the perspectives of parents of young (preteenage) children.^1–4^ These parents have highlighted benefits to remote monitoring glucose data, including reduced stress and anxiety,^1–3^ greater freedom, and improved quality of life for themselves and their child^1,2,4^ and increased opportunities to optimize glucose control.^2^

Data sharing (remote monitoring) is likely to present distinctive issues for older children and their parents, as, from early adolescence, young people vie for autonomy and may not welcome parental oversight.^1,5^ Additional issues may also arise when young people use CGM in conjunction with a closed loop-system as this technology permits followers to access insulin as well as glucose data.

As part of a broader investigation of newly diagnosed adolescents' and their parents' experiences of using closed-loop technology, we explored whether, how, and why, these individuals undertook data sharing (remote monitoring). Our objective was to aid understanding of data sharing practices among parents and adolescents and inform recommendations to support individuals considering data sharing in the future.

## Materials and Methods

Qualitative methods are recommended when little is known about the area being explored as they allow findings to emerge from the data rather than testing predetermined hypotheses. This kind of exploratory approach is vital in studies seeking to generate insights and provide recommendations in line with participants' own experiences and needs.^6,7^ Qualitative studies can also generate hypotheses, which can then be tested out in quantitative studies using large sample sizes.^7^

In this study, interviews were undertaken with adolescents and parents using a topic guide, which contained a list of topics to be covered, rather than a set of predetermined, structured questions. This approach helped ensure the discussion remained relevant to addressing the study aims while affording flexibility for participants to raise issues they considered salient, including those unanticipated at the study outset. Topic guides were developed in light of literature reviews and input from patient representatives and clinical coinvestigators (see Box 1 for information about the main areas explored).

**Box 1. Key Areas Explored in the Interviews**

Parents/caregivers

- Care and support given to the child; role of the parent in supporting diabetes management (e.g., determining insulin doses, carbohydrate counting, monitoring glucose levels, managing hyper- and hypoglycemia).
- Decision-making/negotiation about having access to child's data; (if relevant) views about not being given access to data.
- Frequency and timing of checking and reviewing data; reasons for checking/reviewing data.
- Use of data, for example, when do parents/caregivers intervene after receiving text alerts; types of intervention and reasons for doing so; (any) changes to how parents/caregivers engage with and respond to data over time.
- Perceived benefits and drawbacks of having access/not having access to child's data/text alerts; impact on quality of life and wider family life.
- Experiences of, views about, and reasons for data sharing/text alerts being revoked.
- Information and support needs to optimize effective use of data sharing and minimize any parent–child conflict; guidance and advice to young people and parents who undertake data sharing in the future.
- Any other issues interviewee would like to raise.

Adolescents

- Experiences of living with and managing diabetes in everyday life; (any) worries and concerns about hypo- and hypoglycemia; involvement of parents/caregivers in diabetes management (e.g., determining insulin doses, carbohydrate counting, monitoring glucose levels, managing hyper- and hypoglycemia).
- Decision-making/negotiation about giving parents access to data; reasons for not giving parents access.
- Perceptions and understandings of when, and why, parents check/review data; likes/dislikes of parents having access to this information.
- Experience of, and views about, receiving input and support from parents in response to text alerts/data checking/review; any changes in input and support received over time.
- Perceived benefits and drawbacks of giving parents access to data.
- (if relevant) reasons for revoking access to data.
- Information and support needs to optimize effective use of data sharing and minimize any parent–child conflict; guidance and advice to young people and parents who undertake data sharing in the future.
- Any other issues interviewee would like to raise.

Adolescents and parents were interviewed separately. This approach was informed by our understanding that there could be conflict and disagreement between the two parties^8^; hence, separate interviews permitted interviewees to disclose information they might not wish their parent/child to be privy to. Data collection and analysis took place concurrently so that findings identified in early interviews could be used to iteratively inform areas explored in subsequent ones.

### Recruitment and data collection

Adolescents and their parents were recruited following randomization to a 24-month open-label, U.K.-based, multicenter, randomized controlled trial, followed by an optional 24-month extension phase, which explored the clinical and other benefits of a day-and-night hybrid closed-loop system compared to a multiple daily injection regimen in youth newly diagnosed with type 1 diabetes.^9^ To be eligible for the trial, participants needed to have been diagnosed within the previous 21 days and aged 10–16.9 years. Participants in the closed-loop arm began by using the FlorenceM system, which did not have a CGM data sharing feature (Box 2). During the extension phase, these individuals were moved onto a new iteration of the closed-loop system called CamAPS FX (Box 2), which supports automatic data upload to the cloud at 5–10 min intervals and allows young people to share data with parents/caregivers.

**Box 2. The Closed-Loop Systems and Diasend App Used During the Trial**

FlorenceM hybrid closed-loop system comprised:

- A modified next generation sensor-augmented Medtronic insulin pump 640G (Medtronic, Northridge, CA) with CGM receiver and low-glucose suspend feature.
- Guardian 3 sensor (Medtronic).
- An Android smartphone (Galaxy S4; Samsung, South Korea) hosting the Cambridge model predictive control algorithm (University of Cambridge, Cambridge, United Kingdom) with a propriety translator to allow wireless communication with the insulin pump.

CamAPS FX hybrid closed-loop platform comprised:

- Dana RS insulin pump (Diabecare; Sooil, Seoul, South Korea)
- Dexcom G6 real-time CGM sensor (Dexcom, San Diego, CA)
- An Android smartphone (Galaxy S8; Samsung) running Android 5.0 OS or above, which hosted the CamAPS FX Application incorporating the Cambridge model predictive control algorithm (University of Cambridge) and communicating wirelessly with the insulin pump. Participants could also use their personal smartphone if compatible.
- Real-time upload capability to monitor and share CGM and insulin data to diabetes management system Diasend (Glooko/Diasend, Göteborg, Sweden).CamAPS FX app

In addition to being used to administer mealtime boluses of insulin, the CamAPS FX app included functions enabling users to:

- View a “real-time” graph displaying their sensor glucose levels, rate of insulin delivery, meal-time boluses and carbohydrate intake, high/low glucose range, glucose trend arrows, whether “boost” or “ease-off” functions are activated, and whether the closed-loop was operational (Auto mode on) or interrupted (Auto mode off).
- View summary statistics for daily, weekly, monthly, or three monthly periods, including average glucose, estimated HbA1c, time in/below/above target, number and average duration of hypos, total daily dose/bolus/basal insulin, and percentage of time in Auto Mode.
- Issue instructions to the closed-loop to initiate a “Boost” mode of operation when more insulin was needed (e.g., during periods of inactivity, increased food intake, or during illness or stress), or an “Ease-off” mode when less insulin was needed (e.g., during exercise or when glucose levels tends to be low).
- Receive and personalize alarms/alerts triggered by high/low glucose, and signal loss with the sensor and/or pump, by adjusting the threshold, repeat time and audio sound or vibration, which accompanied an on-screen display, and turn on/off all alerts (except the “Urgent Low” glucose alarm).
- Share (by automatically uploading to the cloud) with health professionals, and their parents/caregivers, near “real-time” glucose levels, insulin, and meal-time bolus data, which could be accessed using the Diasend/Glooko app, and an option to relay alarms activated by users to a user-determined set of “followers” alerting them to high/low blood glucose, sent via SMS texts. To enable data sharing and sending text alerts, individuals could either use their existing phone SIM (if the handset was compatible) or purchase/insert a SIM card to use with the study handset provided.

Diasend app (Glooko/Diasend)

Parents who wanted to view their child's glucose and insulin bolus data in near real-time could download the Diasend app (see: https://play.google.com/store/apps/details?id=com.diasend.diasend&hl=en_US)

CGM, continuous glucose monitor.

Participants were informed about the option for data sharing/remote monitoring when they were switched over to the new system. Instruction (written and verbal) was provided on how parents/caregivers could download the Diasend diabetes management system app (Glooko/Diasend, Göteborg, Sweden) to their own smartphone so that they could review in near real-time (6–12 min lag time), or retrospectively, the young person's glucose levels and insulin boluses (Box 2). These individuals could also be added as “followers” within the CamAPS FX app so they could receive SMS messages when app-generated alarms were triggered. These include alarms/alerts indicating when a user's glucose is above or below user-selected thresholds, or when there is a loss of connectivity between the smartphone and CGM transmitter.

Participants were recruited into the interview study by health professionals from six participating U.K. sites using an opt-in procedure and were interviewed after they had ∼6 months experience of using the CamAPS FX system. This time point was chosen to allow sufficient time for individuals to have become familiar with using the new system in their everyday lives, including the data sharing feature. Purposive sampling was used to ensure diversity in adolescents' age, gender, and parental occupation. Recruitment and data collection continued until we reached data saturation (i.e., until no new findings were identified in new data collected). Interviews were conducted by David Rankin, an experienced qualitative researcher between November 2019 and March 2020. Interviews lasted 1–2 h and were transcribed in full.

The study received approval in the U.K. from Cambridge East Research Ethics Committee (REC ref 15/EE/0324) and the Medicines & Health products Regulatory Agency.

### Data analysis

Interviews were analyzed by Julia Lawton, David Rankin, and Ruth I. Hart (all highly experienced qualitative researchers) using a thematic approach informed by the method of constant comparison.^10^ Interviews were read through repeatedly and cross compared to identify emergent themes and finalize a data analysis plan. This initial period of data familiarization alerted us to striking differences in data sharing practices across the adolescent age range and informed our decision to undertake and report age-based comparisons; it also alerted us to differences in the perspectives and experiences of parents and children within the same dyads. A coding framework was then developed which captured key themes and contextual information needed to aid data interpretation. NVivo11 (QSR International, Doncaster, Australia), a qualitative software package, was used to facilitate data coding and retrieval and coded datasets were subjected to further analyses to allow more nuanced interpretations of the data to be developed.

## Results

Eighteen adolescents (aged 11–18 years) and 19 parents were interviewed. Demographic and other information about the sample are provided in Table 1.

**Table 1.** Demographic Characteristics of Study Participants

Characteristic	*N*	%	Mean ± SD, range
Adolescents with type 1 diabetes (*n* = 18)			
Gender, female	8	44.4	
Age at time of interview, years			14.3 ± 2.1, 11–18
Age breakdown, years^a^			
11–13	6	33.3	
14–16	8	44.4	
17–18	4	22.2	
Duration using CamAPS FX, months			6.3 ± 0.5, 6–7
Parents (*n* = 19)^b^			
Gender, female	15	78.9	
Age, years			48.2 ± 4.49, 40–58
Occupation			
Professional	10	52.6	
Semiskilled	6	31.6	
Unemployed/full-time carer	3	15.8	

^a^ Percentages do not sum to 100% due to rounding.

^b^ One interview included a mother and father.

SD, standard deviation.

Three main themes arose from the analysis, which broadly reflected age-graded variations in whether, how, and when, data were shared and used by parent–child dyads: *remote management; watchful waiting;* and*, being remote*. These age-graded variations were broadly in line with commonly reported adolescent substages, which designate early adolescence as 11–13 years, middle adolescence as 14–16 years, and late adolescence as 17+ years.^11,12^ Broadly, we identified a shift from synchronous practices (parents undertaking *remote management* using “real time” data) to asynchronous practices (parents practicing *watchful waiting* and only offering input and support at some future moment if required) as children got older, with parents of the oldest adolescents simply *being remote*, by virtue of the young person not permitting them access to their data and/or not telling their parent(s) such functionality existed.

These themes are considered in more detail below. While we have separated out our reporting according to the three adolescent substages indicated above, there was some overlap in the practices and experiences reported by participants in the different age groups. There were no apparent differences in participants' accounts according to the young person's gender and parental occupation.

### Remote management

#### Parents' perspectives

All parents of younger adolescents (11–13 years) reported making extensive use of automated text alerts and near “real-time” glucose data accessed via Diasend, especially when their child was asleep or away from home. These parents described checking the latter frequently during the day and/or when they woke at night: “I look at it constantly… Whenever I turn my phone on, to send a text, or to check something I have a quick look.” (002_mum_girl_12yrs). As well as seeking reassurance that glucose levels were in range, parents described using the data to provide “real-time” remote diabetes management support. Most typically, this involved phoning, texting, or calling to the child to make them aware of and issue instructions on how to preempt or address high/low glucose, as soon as a potential problem was identified:

“So, I can be at work and notice if she is perhaps heading for a bit of a hypo, and I can text her and just say, you know, put an ease off on or, you know, just keep a wee eye (open), you're going to need something shortly.” (013 _mum_girl_11yrs)

“sometimes, he's in his room and I have to shout [call], ‘[name] you're high… boost yourself.” (010_mum_boy_13yrs)

Parents regarded these near “real-time” remote management activities as being necessary because of their child's need for practical and emotional support: “She doesn't tend to [check her own glucose readings] unless it's alarmed, if I'm honest… She's 13 so you can't expect her to.” (012_mum_girl_13yrs); “I think (daughter) likes it if I'm a bit more interested, just being aware of the levels and giving her support” (002_mum_girl_12yrs).

When they offered input and support, parents highlighted the benefits of having access to a wider body of information than would have been possible using CGM alone:

“well obviously I've got more of an insight on what his day has been like. I've got an insight on whether he's treating it, or an insight whether he's not putting his carbs in properly, which gives me more information to be able to talk to him about it.” (014_mum_boy_12yrs)

While these parents saw remote monitoring as a parental responsibility, they also noted that their child's willingness to share their data was likely to change as they got older, and, hence, care and effort were needed to sustain positive attitudes for as long as possible:

“You know, children don't want their mother's eye on them too much… At the moment he's a little boy and it's very easy [but] he's growing very quickly and he'll be pushing me away as much as he can…He doesn't seem to resent (data sharing) too much. But I have to be careful… to work with him… to be supportive.” (003_mum_boy_12yrs)

Indeed, some parents noted how there was potential for their son/daughter to feel that their privacy was being infringed because remote monitoring provided information about their activities and behaviors as well as their glucose levels:

“I do wonder if (child's name) will be like, it's a bit of an invasion of her privacy as she gets more into her teen years… because I see she's put carbs in for whatever she's eating, you know, I can see everything she's done, so I am expecting, at some point, that might be a bit of an issue.” (012_mum_girl_13yrs)

Some also expressed worry about their child having relaxed self-monitoring and self-care activities by virtue of their oversight and support. Hence, they emphasized the need to start promoting independence and responsibility:

“he went a little bit lazy because I was checking, because he knew I was checking and, like, I was keeping an eye on it. I said, you still need to have a look you know, I said it's not just me.” (010_mum_boy_13yrs)

#### Early-adolescents' perspectives

Mirroring parents' accounts, early adolescent children also described feeling safer by virtue of having their parents closely monitor their glucose levels:

“It makes me feel a lot more secure, because if I'm not aware of it, I know that my mum will be… Because I'm quite scared of having hypos at certain times, like in assemblies and stuff. And she takes a lot of the weight off my shoulders, because my mum knowing is like another me knowing, so there's never really a problem.” (014_boy_12yrs)

Such individuals also portrayed themselves as not yet ready or able to manage their diabetes without parental input:

“I'll be doing something and I'll look at a message (from Mum) and it'll be like, ‘Put a boost on you're going quite high.’ So I'll be like, ‘Oh yeah, I am.’ And I'll put a boost on.” (010_boy_13yrs)

Hence, these young people were generally very positive about their parents having access to their data and reported feeling more confident and secure by virtue of being closely monitored:

“because it's like if you're in your mother's arms or something like saying like that. You know you're cared for, and you know that em, yeah, you know that you're going to be fine, because my mum is checking what I am like constantly.” (012_girl_13yrs)

### Watchful waiting

#### Parents' perspectives

While parents of middle adolescents (typically 14–16 years) also welcomed opportunities to monitor their child's glucose levels in “real-time,” these individuals described making careful and strategic decisions about whether—and when—to intervene, when text notifications or inspection of Diasend data alerted them to high/low levels:

“for a low, dependent on what the readings are, if it's within the threes, high threes, give it 15 minutes to give him a chance to have some sugar and see.” (015_mum_boy_14yrs)

Indeed, when these parents reflected upon their use of near “real-time” data, they highlighted the importance of practicing watchful waiting, rather than stepping in immediately to offer input. As such parents noted, this approach allowed them to encourage and support their child's autonomy, while enabling them to act as a safety net if a problem such as severe hypoglycemia or disconnection of an insulin infusion cannula had gone undetected:

“It's allowed me to give him more independence. It means you can allow him to deal with things, and then, say, for instance, I can see him going low and I can see he's done nothing about it, then I can get involved, you know, you can give someone that window to deal with it themselves.” (006_dad_boy_14yrs)

A couple of parents, however, reported only fully appreciating the importance of this restrained approach after their eagerness to offer input had resulted in angry responses, and, in 011_mum's case, to her son stopping her from receiving text notifications:

“I was messaging him saying I see you're going low, make sure you put some carbs in… So of course, that really annoyed him, because he was a bit like, ‘why are you doing this? I know, I know, you don't need to send me a message to remind me.’” (011_mum_boy_16yrs)

Most parents also noted how their confidence and ability to practice watchful waiting had been facilitated by knowing that the closed-loop would increase insulin delivery in the event of high glucose or, cut it off if glucose levels started to drop too low:

“if it sends me an alert saying she has an urgent low soon [low glucose alarm], I look at on the phone and see how quick it's coming down and, often it will be enough just to, you know, her basal will have been knocked out by the [closed-loop] for a while, so that might be enough for it to just start coming back up, without needing to do anything. So I can watch and wait.” (017_mum_girl _15yrs)

Parents also endorsed a watchful waiting approach when they reviewed retrospective data via Diasend. Most described undertaking regular (sometimes daily) review of these data: “just to see what he's done during the day, to see where he's bolused and where he's not” (018_mum_boy_15yrs). However, they also noted how they would only speak to their son/daughter if a distinctive and enduring pattern of problematic behavior had become apparent, such as when they regularly failed to administer a bolus dose at lunchtimes:

“If they have a morning break he tends to eat his lunch then. And on a couple of occasions he hadn't bolused for it.. Or he'd forgotten and done it later… Now sometimes when there's a little spike and it's just come back down, you think: that's fine, that's not a problem. But if it was something where you could see a habit forming and it's just not good, you know not a good habit to get into, then I would mention it.” (005_mum_boy_15yrs)

When they did feel it was necessary to intervene, parents described how they tried to introduce their concerns at carefully timed moments and often in relationship to more general questions about how their child was feeling and doing. Indeed, rather than *alerting* their son/daughter to a problem and *instructing* them how to address it, these parents described adopting a *confirming* and *prompting* approach geared toward encouraging their mid-adolescent child to reflect and decide on whether, and what, action was needed:

“if she's been high for a while or she's been low for a while, I would often phone her and just say, ‘I don't know if you've noticed yet, but… you've been high for a little bit or you've been low for a little bit. But that would be it. It's like, you know, and then, are you okay?’ Just, it's sort of a little prompt rather than a full discussion and then it's down to her.” (016_Mum_girl_14yrs)

To mitigate potential conflict, parents also highlighted the importance of communication and getting mutually agreed ground rules in place for how, and when, data should be used:

“I think you need to set out some ground rules with your child… I think it's about, think about how you are going to work with this with your child, because if it only ever becomes a source of conflict with them, actually it isn't particularly helpful, is it? But if it's there as a tool to help you all—so I think it's about thinking about how you would talk to your child initially about how you're going to operate with this…. I think there isn't a one size fits all. I think it's a tool and I think you need to think about how it's gonna help you and your child.” (018_mum_boy_15yrs)

#### Middle adolescents' perspectives

Middle adolescents were mostly positive about their experiences of sharing data, in large part, because they recognized that their parents only stepped in and offered strategic advice when it was really needed and in ways which did not feel overly intrusive or directive:

“because they, I think they're quite sensible with how they use it, if that makes sense. Obviously, they're adults so they are sensible, but I don't think they're like overbearing or anything with it. I think they just, they look at it to check if I'm okay, and then that's it…it's not really often that I'll ever get a text saying, check your blood sugar, are you okay? I think they use it quite sparingly, only if they feel they need to.” (016_girl_14yrs)

Indeed, it is noteworthy that most of these young people seemed unaware of how frequently their parents were checking and reviewing their data, with some speculating that this only happened on a weekly or monthly basis.

Middle adolescents also noted how data sharing enabled their relationships with their parents to become more normalized, as their conversations were no longer dictated by diabetes and requests to provide information about their glucose levels as parents were now able to access this information on their own smart devices:

“And they ask questions when they need to, rather than all the time. So instead of when I get home, ‘oh have your blood sugars been fine’, it's, well, they already know because they can check the app… Because sometimes it could get a bit frustrating, having my day focused around my blood sugar and then having them ask about it on top of it.” (018_boy_15yrs)

Middle adolescents also reported an increased ability to go out and socialize without excessive (unwanted) parental interference, as parents were now able to see for themselves that glucose levels were in range, or that they (or the closed-loop system) had taken action to address out of range readings:

“If I went away on a school trip or something, they don't have to text me and check is everything all right, they can see. Which is actually a good thing, them being able to see the app, because they can now check it without like texting or disturbing me.” (005_boy_15yrs)

Notably, most middle adolescents indicated that their initial motivation and on-going willingness to allow parents access to their data had been driven by a desire to lessen their parents' own anxieties:

“I was fine with it [allowing parents to access data via Diasend] because it's more for them rather than me, I know I'm fine to look after myself the majority of the time, but it's just so they know I'm doing okay… It makes them feel better. And then that, in turn, just makes it easier for me.” (018_boy_15yrs)

For this reason, while middle adolescents mostly welcomed parents being able to access their data via Diasend, they were more ambivalent about them receiving text alerts. As these individuals noted, text alerts could cause parents needless worry, because they felt they were well capable of responding to alarms alerting them to high/low glucose levels themselves:

“If you got an alarm saying I was low, I could just be going low, it's not like a worry, I would already have sorted it out, so something like that he [father] doesn't always need to worry about.” (006_boy_14yrs)

### Being remote

#### Parents' perspectives

Parents of late adolescents (16+ years) generally appeared unaware of data sharing opportunities until asked about them in their interviews. These individuals noted how their son/daughter had generally chosen to attend appointments alone, including the appointment where they were moved onto CamAPS FX system, and how they had rarely been made privy to any discussions which had taken place. These parents also indicated no surprise that their son/daughter had not made them aware of the data sharing feature or given them opportunities to access their data, due to their son's/daughter's desire to manage their diabetes independently:

“we didn't know that (remote monitoring) was an option, but I mean, I know what his response to that would be, which is that it's none of my business… He doesn't appreciate any questions or involvement at all.” (007_mum_boy_17yrs)

Such parents also noted how, because of the child's age and developmental maturity, they needed to be respectful of their right to privacy and autonomy:

“if she was younger I'd probably insist on having it on my phone. Because she's at that age now, she goes out, she has her own freedom, so it's giving her more responsibilities for her own health.” (001_dad_girl_16yrs)

They also suggested that, because their support was no longer needed or wanted, having access to the child's data could be experienced as “really intrusive, like we are spying on them.” (008_dad_girl_18yrs)

#### Late adolescents' perspectives

Mirroring parents' accounts, late adolescents described seeing themselves as fully responsible for and able to manage their diabetes independently; hence, they noted how parental involvement, including oversight of their data, was not needed or wanted:

“I'm always on my phone, so I'm always going to know what my bloods are.. And I think if my mum and my dad did have it, I think they would start like: oh (child's name), you're going to go low, or you're going to do this. But I know already. And that'd just annoy me” (001_girl_16yrs).

Indeed, many of these individuals noted how they had cut their parents out of their self-management loops entirely: “if I have an issue, I just call (diabetes nurse), I don't bother talking to my parents.” (008_girl_18yrs), especially those who had left home (e.g., to attend university). As well as wanting and expecting their own independence, these individuals noted how data sharing would serve no practical purpose and, furthermore, could cause parents unnecessary anxiety: “because if they could see my data and see a problem, there was nothing they could do about it.” (007_boy_17yrs).

## Discussion

This study has explored parents' and adolescents' experiences of data sharing in the context of using a closed-loop system. In keeping with studies which have highlighted age-graded variations in how these individuals undertake and share diabetes management tasks,^11^ we observed a transition from synchronous/interventionist approaches to asynchronous/less interventionist practices as young people transitioned toward adulthood.

Specifically, while parents of younger adolescents reported making extensive use of “real-time” data to remote manage their child's diabetes, those of middle adolescents described practicing watchful waiting and only intervening after a pattern of problematic behavior became apparent or concerns about their child's safety arose. In the oldest age group, parents were simply remote because their involvement in diabetes management was no longer welcomed and considered helpful; hence, none of these individuals were permitted access to their son/daughter's data.

We have also drawn attention to differences in adolescent and parental understandings of the purpose and value of data sharing. While parents emphasized opportunities to support their child and help them optimize their glucose control, middle adolescents highlighted motivations and agendas which were primarily psychosocial. These young people emphasized that data sharing was done primarily for the benefit of the parent. However, they also noted benefits to themselves. These included increased opportunities to go out and socialize without parental interference and more normal interactions with parents as these were not dominated by requests to disclose information about their glucose levels.^13–16^

Various commentators have highlighted the importance of parental involvement during the adolescent period, as this can improve adaption to diabetes and glycemic control,^17–19^ while family conflicts can act as barriers to adolescents achieving optimal glycemic control.^20,21^ Hence, the importance of building positive family involvement has been emphasised.^15,18^ While younger participants and their parents reported data sharing to be unproblematic, we have highlighted potential for conflict to arise as children got older and wanted greater autonomy. Not only did parents of maturing adolescents note the importance of using the data in careful and strategic ways, which respected their child's autonomy, participants also indicated potential for annoyance and for parental data access to be revoked if parents offered unwelcomed and/or excessive input.

In keeping with Litchman's recommendations,^4^ these observations underscore the need for clear ground rules to be established and agreed before data sharing commences. To do this, the diabetes care team, including a diabetes specialist nurse, could encourage and support adolescents and their parents to discuss, negotiate, and clarify how, and in what situations, the latter will use data to offer input and support.^22^ In doing so, it may also be helpful for health professionals to assess family dynamics and where families are on the spectrum from a parent-dominated to an adolescent-dominated diabetes management pattern.^22^

As our findings further suggest, regular review of ground rules may be necessary due to the parent-child relationship being in a constant state of transition. In addition, consideration could be given to practices which appeared to work well for parent–child dyads during the mid-adolescent phase, including parents adopting confirming and prompting rather than directive approaches when concerns about their son/daughter's diabetes management arose.

Alongside the need for ground rules, customized, tailored designs/options are an important consideration, as Bedrossian et al.^23^ have recommended more generally in the design of information technology to support type 1 diabetes management among adolescents. Indeed, our study has highlighted considerable variability in data sharing practices, with parents of younger adolescents, in particular, valuing technology which permitted quick and easy access to data in “real-time,” while those of middle adolescents made greater use of retrospective data, including data offering insight into behavioral patterns, which could be detrimental to long-term glycemic control. Hence, to optimize acceptability and effective use, having options to use data in different ways and to allow data use to change over time, should be included and/or retained in future iterations of the technology.

A key study strength was the inclusion of adolescent as well as parental perspectives, as this enabled us to identify and explore differences in how each group perceived and valued data sharing. An additional strength was our decision to interview adolescents of different ages, although use of a longitudinal design would have permitted better exploration of how data sharing activities change over the adolescent developmental period.

Our study examined experiences of data sharing in the context of using a closed-loop system; this might limit the generalizability of some of our findings. Indeed, arguably, one of the parental styles reported in our study—watchful waiting—may have been facilitated and enabled through using a technology which automatically addresses high/low glucose levels. We have also highlighted the potential for heightened concerns about privacy to arise by virtue of parents having access to more data than would have happened had their son/daughter used a CGM sensor alone, rather than CGM technology in conjunction with a closed-loop system.

When taking our findings and recommendations forward, readers need to consider that interviewees were involved in a clinical trial and, hence, may have been particularly motivated and engaged. Participants were also very newly diagnosed when they were recruited into the trial and had no experience of using diabetes technologies apart from the two closed-loop systems investigated in the trial. Future research could consider use of longitudinal designs that would permit the same parent–child dyads to be followed-up over time and/or quantitative designs with larger sample sizes to establish whether our findings are generalizable to a wider adolescent diabetes population.

## Conclusion

In conclusion, our study has highlighted considerable variability in how data sharing was perceived, valued, and used by parents and adolescents of different ages. To help parent–child dyads use data sharing/remote monitoring in ways which minimize conflict and optimize constructive parental support, we recommend tailored input and support, which takes account of family dynamics, the young person's developmental maturity, and the different ways in which data are used across the adolescent age range.
